# Preparation and Characterization of Graphene Oxide for Pb(II) and Zn(II) Ions Adsorption from Aqueous Solution: Experimental, Thermodynamic and Kinetic Study

**DOI:** 10.3390/nano10061022

**Published:** 2020-05-27

**Authors:** Carlos A. Guerrero-Fajardo, Liliana Giraldo, Juan Carlos Moreno-Piraján

**Affiliations:** 1 Departamento de Química-Grupos de Investigación Aprena y Calorimetría, Facultad de Ciencias, Universidad Nacional de Colombia-sede Bogotá, Cra. 45 No. 26–85, Edificio 451, Bogotá 111321, Colombia; caguerrerofa@unal.edu.co (C.A.G.-F.); lgiraldogu@unal.edu.co (L.G.); 2 Facultad de Ciencias, Departmento de Química, Universidad de los Andes, Bogotá 111711, Colombia

**Keywords:** Graphene oxide, isotherms, metallic ions, thermodynamic study

## Abstract

A thermodynamic and kinetic study of the adsorption process of Zn (II) and Pb (II) ions from aqueous solution on the surface of graphene oxide (GO) to establish the mechanisms of adsorbate–adsorbent interaction on this surface. The effect of pH on the retention capacity was studied and adsorption isotherms were determined from aqueous solution of the ions; once the experimental data was obtained, the kinetic and thermodynamic study of the sorption process was carried out. The data were fitted to the Langmuir, Freundlich, Dubinin-Raduskevich and Temkin isotherm models. The results showed that Zn(II) and Pb(II) on the GO adsorbing surface fitted the Langmuir model with correlation coefficients (R^2^) of 0.996. Kinetic models studied showed that a pseudo-second-order model was followed and thermodynamically, the process was spontaneous according to the values of Gibbs free energy (ΔG^o^). N_2_ adsorption isotherms were determined and modeled with the NLDFT (nonlocal density functional theory) and QSDFT (quenched solid density functional theory) kernels.

## 1. Introduction

Nowadays environmental pollution is increasing due to the so-called “industrial development” and the proliferation of small and large industries that produce a large amount of pollutants. Within this type of industry can be mentioned mining, smelting, electroplating and battery manufacturing, all of which generate high pollution in lagoons, rivers, lakes and finally the oceans, due to the amount of deposits of heavy metal ions [1,2,3]. This generates serious health problems worldwide, due to the ingestion of these pollutants. Metal ions produce a negative impact due to their toxicity to animals in the water, and later, through the food chain, they pass to humans.

It is important to develop techniques and methods that allow efficient removal of metal ions from aqueous solutions. Several of the methods, which have been designed, use porous-type materials due to the control that can be exerted during their synthesis since they can be functionalized to obtain special characteristics.

Nanomaterials, due to their special characteristics, such as high specific surface area, high number of sites that allow binding with the adsorbates that need to be adsorbed, abundant functional groups and adequate pore size, are considered desirable adsorbents for the treatment of wastewater contaminated by heavy metals [1,4,5].

Porous materials reported and used successfully to remove metal ions are, for example, activated carbons, activated carbon fabrics, SBA-15, aerogels and carbon xerogels and, recently, the metal organic frameworks (MOFs). However, within the group of adsorbent nanomaterials, various techniques to synthesize and develop new solids continue to be investigated.

A suitable solid for the adsorption of metal ions, which has been investigated in recent years, is graphene oxide (GO), which is characterized by having as characteristic groups -C=O and -C-OH in its basal plane and -COOH at its edge [1,5,6]. GO is a sheet of graphene functionalized with different oxygenated groups, which used as a precursor to synthesize graphene or as a graphene material itself. In general, the GO contains carboxyl, lactone, phenol, carbonyl, anhydride, ether, quinone and epoxy groups [7,8,9,10,11]. Some authors have also reported the presence of sulfur-containing functional groups for GO due to the presence of H_2_SO_4_ impurities during synthesis [12,13,14,15,16,17,18,19]. It is insulating, hygroscopic, with high oxygen content, and very hydrophilic. Several investigations have shown that graphene oxide (GO) has good adsorbent properties, particularly in solid–liquid systems, which is attributed to the magnitude of the developed specific surface area, the amount of surface groups with oxygen content and the excellent dispersion characteristics [1,12,13,14,15].

The adsorption capacity of an adsorbent depends on a series of properties and, logically, on the combination of them: (1) the intrinsic properties of the adsorbents, origin of the adsorption material, specific surface area, structure and distribution of pores and the type and quantity of functional surface groups; (2) the physicochemical properties of the adsorbate and (3) the environmental conditions under which it will be applied [1].

For the adsorption of metal ions on GO, the properties of both GO and the characteristics of metal ions are the fundamental factors for establishing the adsorption performance in investigations of this type. Recent publications show that GO has the ability to adsorb metal ions, including Na (I) [1,16], Cd(II), Co(II) [1,12,17,18,19,20,21,22,23,24,25,26,27,28,29,30,31,32,33,34], Cu (II) [1,17,35,36], Pb(II) [1,4,19,20,37,38,39], Ni (II) [1,4], and Zn (II) [1]. However, despite the amount of research and publications carried out so far, detailed studies of ion adsorption on GO do not especially contemplate the analysis of adsorption mechanisms, as well as adsorbate–adsorbent thermodynamics and the correlation between the characteristics of ions and GO.

In this research, GO was synthesized from graphite (Gr) using methods reported in the literature [16,17,18,19,20,21,22,23,24,25,26,27,28,29,30,31,32,33,34,35]. Graphene oxide was characterized by a variety of techniques to ensure that this material was synthesized. Other techniques as N_2_ adsorption isotherms at 77 K were determined to establish the textural characteristics and pore model using the NLDFT (nonlocal density functional theory) and QSDFT (quenched solid density functional theory) kernels. Then, GO was used to perform the studies of adsorption capacity from aqueous solutions of Pb(II) and Zn(II) ions. The adsorption kinetics and thermodynamics in the solid–liquid interface were also evaluated by applying different models.

## 2. Materials and Methods

### 2.1. Materials and Reagents

#### Synthesis of Graphene Oxide

**Figure 1:**
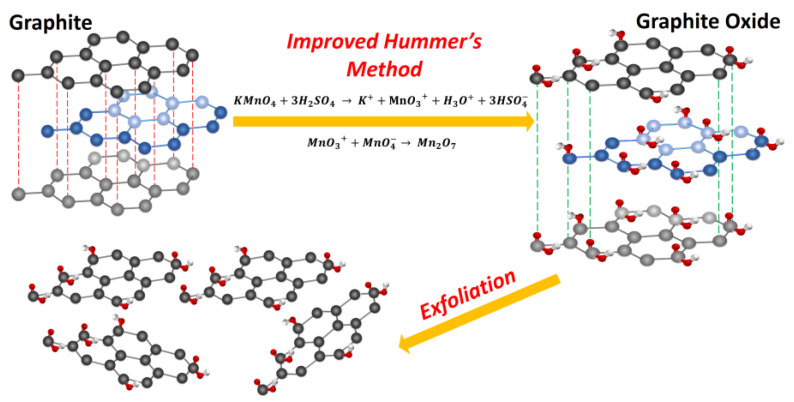
Scheme of the preparation of graphene oxide from graphite.

Hummers and Hoffman, in 1958, [40,41] proposed one of the most widely used methods for the synthesis of graphene oxide (GO), which consists of the oxidation of graphite to graphite oxide and subsequently an exfoliation to GO [22,23,24,25], as shown in Figure 1.

GO was prepared from graphite sheets (Flakes, Sigma-Aldrich, St. Louis, MO, USA, CAS 7782-42-5) using the improved Hummers method reported in the literature and taking into account changes that have been suggested by several authors to perform a procedure with good performance and laboratory safety [16,17], in which the main process consists of oxidation. KMnO_4_ (18.0 g, equivalent weight –equ/wt-) (Sigma-Aldrich, CAS 10294-64-1) was slowly and carefully added in six equal portions, to a mixture in 9:1 ratio of H_2_SO_4_/H_3_PO_4_ (360:40 mL) (concentrated acids, Sigma-Adrich CAS 7664-93-9(H_2_SO_4_) and Sigma-Aldrich CAS 7697-37-3 (HNO_3_)) previously prepared, and graphite flakes (3.5 g, 1.17 wt equiv). The process generated an exothermic reaction, therefore the temperature was strictly controlled, so that it did not exceed the range of 35–40 °C. Subsequently, the reaction system was heated to 50 °C and stirred for 36 h. After 24 h had elapsed, the mixture was naturally cooled to room temperature and then carefully brought to a temperature of 1 °C using a cryostat with which adequate temperature control was achieved. A volume of 1.5 mL of H_2_O_2_ (30%, Sigma-Aldrich CAS 7722-84-1) was added dropwise in order to remove KMnO_4_ excess. The mixture subsequently was adjusted to pH 1 by the addition of deionized water (3.0 × 10^−7^ ohm^−1^ · cm^−1^, Lab Manager ™). The resulting solution was centrifuged (Universal Z 326K Refrigerated Centrifuge, Hermle ™Brand, Wehingen, Germany) at 7000 rpm for 20 min until separation of the solid from liquid. The precipitate was then washed with deionized water, HCl (36.5–38%, Sigma-Aldrich, CAS 7647-01-0) and ethanol (99.5%, Sigma Aldrich) consecutively, five times, and then coagulated with diethyl ether (99%, Sigma- Aldrich, CAS 60-29-7). This last solvent was removed by heating carefully the solution at 45 °C. GO was then exfoliated with ethanol using an ultrasound bath (Fisher Brand, Model CPXH, Boston, MA, USA) for 90 min. Finally, the GO was dried at 80 °C for 24 h and ground in an agate mortar, until passing through a 100 mesh.

### 2.2. Graphene Oxide Characterization

Once the graphene oxide has been synthesized, it is subjected to a series of analyses with the objective of establishing whether the oxidation from graphite was effective. For this, various techniques were used that were applied to both graphite (Gr) and graphene oxide (GO), to establish the structural changes that were made to the graphite to convert it to graphene oxide. The Zeta potential was measured using a Horiba SZ-100Z. The morphology was analyzed by scanning electron microscopy (SEM) using Jeol, JEM 2100 model (Jeol, Tokyo, Japan) unit with an accelerating voltage of 200 kV. Transmission Electron Microscope (TEM) images of the adsorbents were collected on a JEM-2100F transmission electron microscope (Jeol, Tokyo, Japan). Fourier transform infrared spectrophotometry (FT-IR; Thermo Scientific inc., Nicolet iS10, Boston, MA, USA) and X-ray diffraction (XRD; Malvern Panalytical’s, X) were used to investigate the samples from the structural point of view. Pert3 MRD, 40 kV/100 mA X-ray ((λ = 0.15418 nm) step size 0.02°). A UV–Vis spectrometer (UV–VIS; Hitachi inc., U-3900. Tokyo, Japan) equipped with a reflectance diffuse cell was used to measure the optical absorption properties of Gr and GO to verify the presence of the C=O and C=C chromophores characteristic of these samples. For the experimental procedure 2.5 mL of each sample were taken at a concentration of 0.05 mg/mL in ethanol, and these were deposited in a quartz cell. The analysis was performed in a UV-visible spectrophotometer.

To establish the thermal stability of the samples, a thermogravimetric analyzer (TGA; Perkin Elmer Inc., STA6000, Boston, MA, USA) used; the analysis was conducted under nitrogen atmosphere (150 mL min^−1^) with a heating rate of 5 °C·min^−1^. The chemical states of the composition of elements of GO and Gr were investigated following the procedure recommended in the scientific literature [40] by means of X-ray photoelectron spectroscopy (XPS) using the ThermoFisher ESCALAB 250i system (Boston, MA, USA). The binding energy values of the XPS lines were calibrated using the adventitious carbon C 1 s peak at 284.8 eV as reference.

The Raman spectra of the solids recorded using a LabRam HR800 UV microscope-spectrometer (Horiba Jobin Yvon, Tokyo, Japan) (5 mW argon laser excitation with 514.5 nm wavelength and 50× Olympus lens). At least four spectra were acquired for each sample. OriginLab software was used for curve fitting. Samples from 1000 to 3000 cm^−1^ were scanned to visualize and analyze bands D and G. It is necessary to mention that Raman spectroscopy allows the study of materials from the graphene families. This technique provides information on the degree of graphitization and the defects of the sp^2^ carbon structure in the samples.

### 2.3. Analysis of N_2_ Adsorption Isotherms at 77 K

Nitrogen adsorption-desorption isotherms at −196 °C for graphite (Gr) and graphene oxide (GO) were taken using IQ2 sortometer (Quanthachrome Inc, Boyton Beach, FL, USA).

Before starting to determine the adsorption isotherms, the samples were degassed at 250 °C and under vacuum of 10^−5^ mbar for 5 h to eliminate all adsorbed species that could interfere with the measurements. The surface area was determined using the Brunauer–Emmett–Teller (BET) equation [42,43,44,45], using the linearity criterion in the P/P° range of 0.05–0.35, while the Dubinin–Radushkevich methods (DR) [46,47,48,49] and functional theory of density (DFT) [28,29,30,31,32,33] were used to complete studies of textural properties. In particular, through the equation DR it was possible to calculate the volume of micropores DR, V_o_, and the average pore width, L_o_, was evaluated by the following expression [50,51,52,53,54,55,56]: L_o_ = 10.8/(E_o_−11.4), where E_o_ is the energy characteristic obtained by applying the DR equation to experimental data. The equation was applied in the relative pressure range 2 × 10^−6^ ≤ P/P° ≤ 0.2. The pore size distribution (PSD) was calculated using the methods corresponding to the theories of the non-local density functional theory (NLDFT) and the quenched solid density functional theory (QSDFT) assuming cylindrical (cyl.), slit and combined-shape models, cylindrical and slit (cyl.-slit) for the pores of the solid tested (Gr and GO) [56,57].

### 2.4. Adsorbate–Adsorbent Interaction Studies

#### 2.4.1. Zn (II) and Pb (II) Ions Adsorption Capacity in Batch on GO

GO-metal ion adsorption studies, batch experiments carried out from aqueous solution under constant agitation, for the ions used as adsorbates: Pb (II) and Zn (II). Twenty milligrams of GO were taken, which were placed in 20 mL of a solution containing each of the respective adsorbates Zn (II) and Pb (II)) in a concentration range between 2 and 100 mg L^−1^. The contact time of adsorption for both Zn (II) and Pb(II) on the GO surface was established by performing preliminary experiments, and from the obtained results it was determined that 210 min were sufficient to reach the balance between GO and the respective ion solutions. After each adsorption experiment, the containers containing the solutions were placed in an ultrasound bath for 5 min at pre-established temperatures. Subsequently, the respective Pb (II)-GO and Zn (II)-GO solids were filtered using a 0.1 µm cellulose membrane filter and the suspensions containing GO-ion were further centrifuged at 6000 rpm for 5 min. The remaining concentrations of Pb(II) and Zn(II) were then analyzed using AAS (Perkin–Elmer Analyst 700 (Boston, MA, USA) (±0.01%))—atomic absorbance spectrophotometry [30,31,32,33,34,35,36,37,38,39,40]. The amount of adsorbed of the respective ion by the GO was calculated by the difference between the initial and residual concentrations determined by AAS of the respective adsorbate in the aqueous solution. The adsorption capacity of each ion on the GO was calculated using Equation (1):

(1) $$q_{e}=\left(\frac{C_{o}-C_{e}}{W}\right)\times V$$

where q_e_ corresponds to the amount of Pb(II) or Zn(II) adsorbed by the adsorbent (mg·g^−1^) (GO), C_o_ refers to the initial concentration of Pb(II) or Zn(II) (mg·L^−1^), Ce is the concentration of the ions (mg·L^−1^) after the adsorption process, W is the adsorbent mass (g) and V is the volume of the ion solution (L) [58,59,60,61].

#### 2.4.2. Kinetic and Thermodynamic Study of the Adsorption of Zn(II) and Pb(II) Ions on Graphene Oxide

The design of the experiments to carry out the adsorption kinetics studies was carried out by adding 20 mg of GO as an adsorbent in aqueous solutions of Pb (II) and Zn (II) (20 mL) of a fixed concentration of 60 mg L^−1^. After a certain period of time (10–80 min), the samples were collected and the concentration of Pb(II) and Zn(II) in the aqueous solutions was established using the AAS atomic absorbance spectrophotometry technique (Perkin–Elmer Analyst 700, Boston, MA, USA, (±0.01%)).

The adsorption capacity of Zn(II) and Pb(II) at time t (q_t_), in mg^−1^, was calculated using Equation (2) [62]:

(2) $$q_{t}=\left(\frac{C_{o}-C_{t}}{W}\right)\times V$$

where, C_o_ (mg L^−1^) is the initial concentration of each ion and C_t_ (mg L^−1^) is the concentration of Zn(II) or Pb(II) at time t, V is the volume (L) of the solution of the respective ion and W is the adsorbent mass (g).

Finally, experiments carried out at different temperatures: 298 K, 308 K and 318 K, to realize the respective thermodynamic investigation, using some mathematical models.

## 3. Results

### 3.1. Characterization of Adsorbates

**Figure 2:**
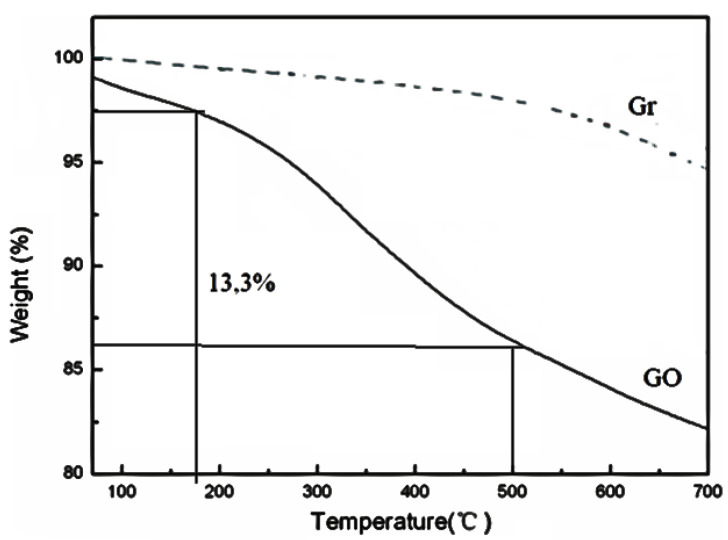
Thermogravimetric analysis (TGA) curves of graphite and graphene oxide.

Figure 2 presents the results obtained for the graphite and graphene oxide (GO) samples, prepared in this investigation, using the thermogravimetric analysis (TGA) technique. The TGA curve for graphite in its pure form shows a weight loss of around 3.5% in the studied temperature range (maximum at 700 °C). The TGA of graphite changed at around 550–650 °C presenting a slight weight loss of 3% that corresponded to generation of CO and CO_2_.

If the thermal behavior of Gr is compared with that obtained for GO in these TGA studies, according to what is shown in Figure 2, it is clear that the thermal stability of GO is less. Some authors have proposed that this fact is due to the instability of Van der Waals forces in the GO as a function of temperature [63,64]. If the TGA obtained for the GO is analyzed in detail, there are interesting results that are worth highlighting. At the beginning of the TGA for GO, a loss of mass is observed as a function of temperature, which according to the scientific literature is associated with the decomposition of labile functional groups that contain oxygen, producing CO, CO_2_ and steam. This weight loss for GO was calculated and turned out to be approximately 13.3%, in the temperature range of 185–500 °C. The TGA shows a weight loss close to 2% below 100 °C attributed to the removal of nonstructural adsorbed water [63,64].

**Figure 3:**
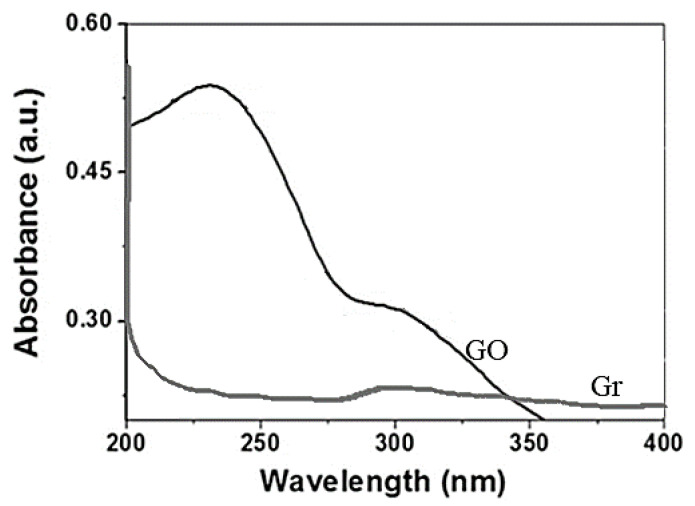
UV–Vis spectra corresponding to Gr and GO samples.

Figure 3 shows the results obtained using the absorption spectroscopy technique in the ultraviolet–visible range (UV-Vis) for the Gr and GO samples. GO had a very pronounced band at 235 nm that was due to the transition of the π-π * electrons within the C-C aromatic bond of the graphene layers, while an additional shoulder-like band, located at 306 nm, was associated to the n-π * transition, due to the presence of oxygen-containing groups according to several authors [65,66,67].

The UV-Vis spectrum corresponding to Gr did not have protruding bands; the absorption spectrum was practically a line with a slight band at 270 nm; n-π * transitions could not be associated, since the UV-Vis spectrum did not reflect them [68,69,70,71,72].

**Figure 4:**
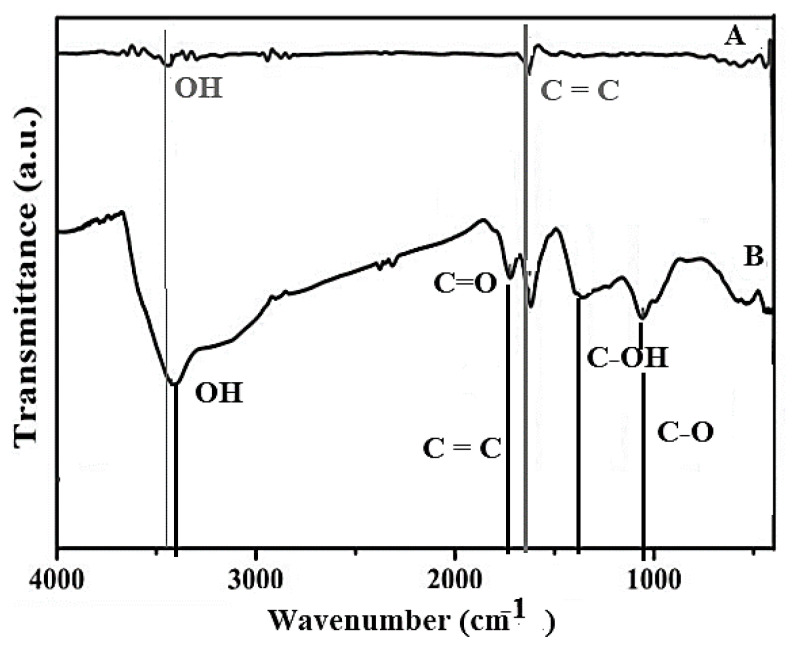
Fourier transform infrared spectroscopy (FT-IR) spectra of A-natural graphite and B-GO.

Figure 4 shows the results obtained for the Gr and GO, corresponding to the analysis by infrared spectroscopy (FT-IR), which allows establishing the characteristic bands of the graphitic and graphene groups, such as hydroxyls, carbonyls, among others [68,69,70,71]. In Figure 4, the FT-IR spectrum corresponding to natural graphite (marked as line A), presents a characteristic band located at ~1640 cm^−1^ (band C=C) that corresponds to skeletal vibrations of the graphite domains, and also shows a band in ca. 3440 cm^−1^ [68,69,70,71,72], which is usually assigned to the OH stretch corresponding to the absorbed water molecules. When the oxidation process was carried out to obtain the GO, the FTIR shows the appearance of a band towards 1695 cm^−1^, due to vibrations of the -COOH type (C=O in carboxylic acid), which is a clear indication that the graphene oxide, GO, was successfully synthesized (Figure 4, line B) [68,69,70,71,72]. Other bands that corroborate the successful preparation of the GO are: the band that appears towards ~1390 cm^−1^ that corresponds to the C-OH group, which together with the band to ca. 3470 cm^−1^, is assigned to the OH stretch mode corresponding to the -COOH groups and the -OH groups attached on the GO surface. Additionally, a band was presented towards ~1075 cm^−1^, which is due to the vibrations of the CO groups in the functions of the carboxylic acid or epoxy groups on the GO surface [68,69,70,71,72] as well as the C=O vibrations around 1675 cm^−1^.

The results corresponding to X-ray diffraction (XRD) allow us to establish the changes that occur in the morphology of each of the samples that used in this investigation: Gr and GO [73].

**Figure 5:**
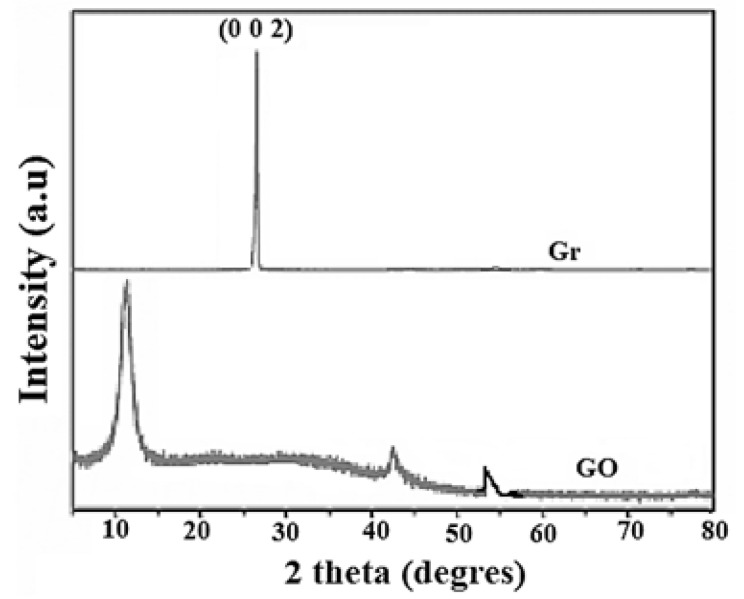
X-ray diffraction patterns corresponding to graphite and graphene.

X-ray diffraction patterns for Gr and GO shown in Figure 5. For Gr there is a clear, well defined, and characteristic peak towards 2θ = 27°, which corresponds to reflection (002) [74].

On the other hand, when performing the GO analysis, this sample has a peak around 2θ = 13.7° which corresponds to the reflection (001) characteristic of GO [75,76,77]. These results corroborate what was found by means of the FTIR analysis, that is, the graphene oxide was obtained from pure graphite [52,53,54]. However, to verify these results, two additional analyses were performed. One of these was to determine the Raman spectra for both Gr and GO synthesized in this work, to investigate possible alterations or defects in the carbon crystal structure.

**Figure 6:**
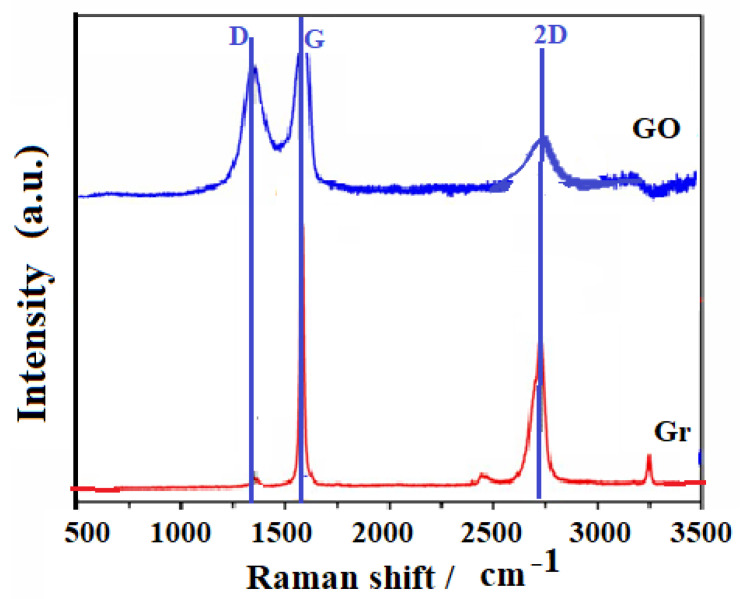
Raman spectra for graphite (Gr) and graphene oxide (GO).

In Figure 6, the presence of the D, G and 2D bands in the spectrum corresponding to the Gr shown. They are presented in this spectrum for the precursor graphite (Gr), bands whose position are approximately at 1350, 1570 and 2740 cm^−1^, respectively. Band G is the results of the vibrations of the sp^2^ carbon atoms in the plane, band D is associated with the presence of structural defects of the Gr and band 2D is a harmonic of band D.

**Figure 7:**
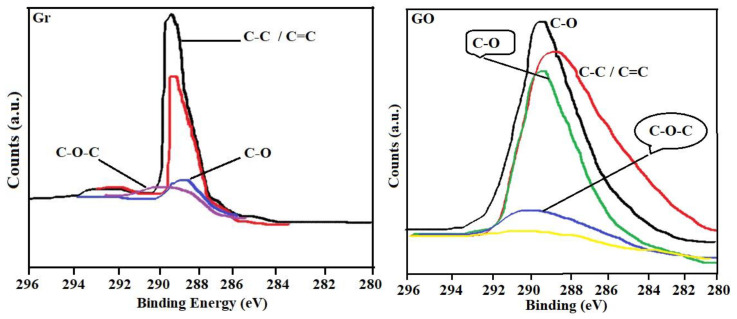
Binding energies of the C 1s levels spectra of Gr and GO. The peaks were deconvoluted into peaks C–C, C–O–C, C=O and COOH groups.

The spectrum for the Gr shows that the solid has an ordered and highly crystalline structure, taking into account the intense and symmetrical G band, a weak D band and the 2D band, which reveals a stacking order. Now if the Raman spectra between Gr and GO that are presented in Figure 6 are compared, the results clearly show the modification that was made on the Gr through the oxidation process to obtain the GO. The GO has a broad D band and medium intensity compared to the G band [77,78,79,80,81]. It is probable that if there are some atoms that are not organized in the GO, these may have generated the increase in band D, which corresponds to the sp^3^ domains, or also if there was a decrease in the size of the graphite crystals, which correspond to sp^2^ domains [77]. This shows that there is a coexistence of sp^2^ and sp^3^ hybridization, that is, that GO contains crystalline and amorphous forms of carbon. However, as seen in Figure 6, the intensity of band D is less than band G, showing that there is a higher proportion of the crystalline phase in GO. The widening of the bands suggests the introduction of structural defects, according to the X-Ray diffraction (XRD) results (see Figure 5). It is clear that the G band does not undergo a noticeable widening, which suggests that there are few defects in the plane. The widening of the D band is due to an increase in the dispersion of the stretching frequencies of the bonds, induced by the incorporation of the functional groups [57,58]. Likewise, a displacement of the 2D band towards low frequencies is observed, indicative of the reduction in the number of layers [78]. When performing an analysis by X-ray photoelectron spectroscopy (XPS) (see Figure 7) for graphene materials such as Gr and GO, it is possible to establish the percentage amount at the atomic level of carbon and oxygen by careful review in the region C1s and O1s [77,78,79,80,81]. In this investigation, 93.7% by weight of carbon and 6.3% by weight of oxygen were obtained for the Gr (this as a function of the atomic concentration); while, for GO, 72.4% of carbon and 27.6% by weight of oxygen were determined. In Figure 7, the C1s signal recorded observed between 296 eV and 280 eV of binding energy for both graphite and GO. When performing an analysis by X-ray photoelectron spectroscopy (XPS) (see Figure 7) for graphene materials, such as Gr and GO, it is possible to establish the percentage amount at the atomic level of carbon and oxygen by careful review in the region C1s and O1s [56,57,58]. In this investigation, 93.7% by weight of carbon and 6.3% by weight of oxygen were obtained for the Gr (this as a function of the atomic concentration); while for GO 72.4% of carbon and 27.6% by weight of oxygen were determined. In Figure 7, the C1s signal recorded was observed between 296 eV and 280 eV of binding energy for both graphite and GO.

The Gr has contributions of sp^2^/sp^3^ and in addition to oxygenated functional groups such as hydroxyls and ethers (C–O, C–C, C–O–C and C=C). The GO presents a characteristic band corresponding to C–O, greater than that observed for graphite, which is due to the increase of oxygenated groups found in the graphitic structure, resulting from the oxidation process during its synthesis from Gr [82,83].

**Figure 8:**
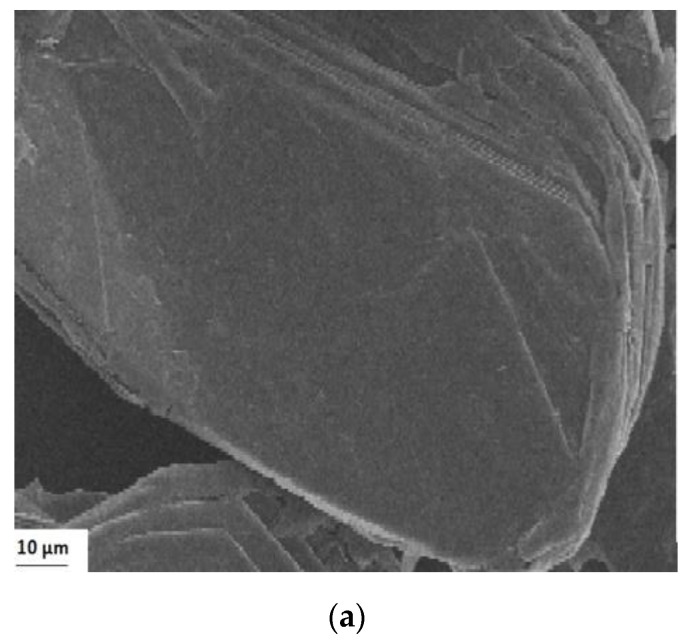

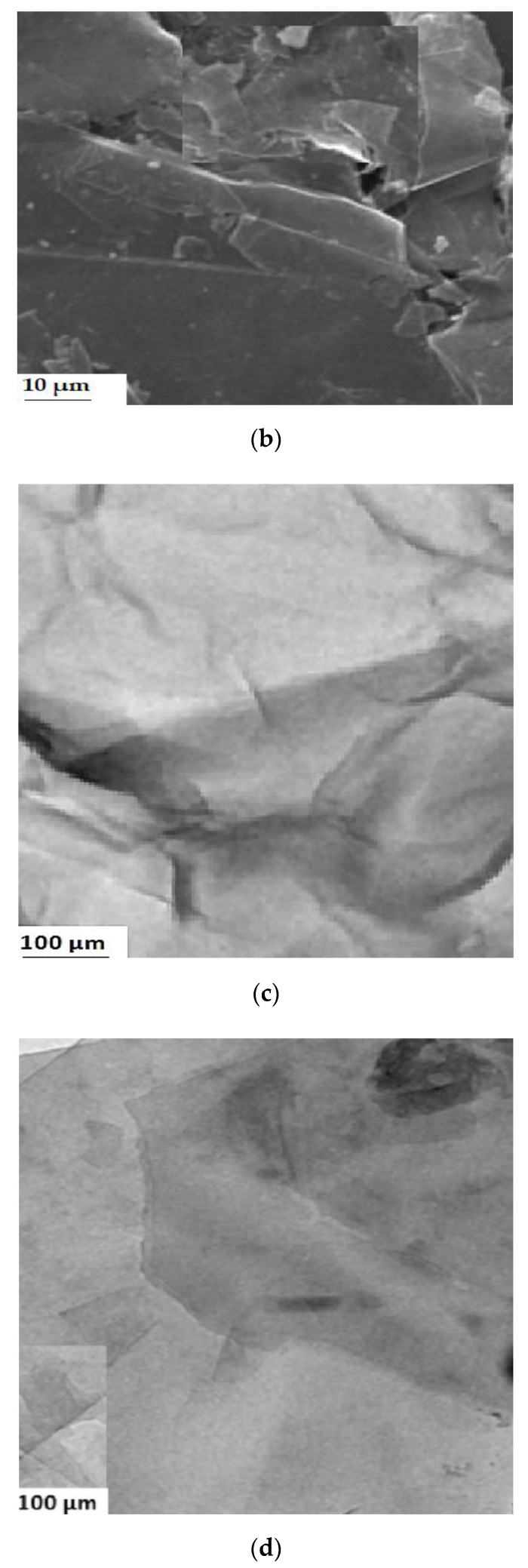
Scanning Electron Microscopy (SEM) of (**a**) Gr (**b**) GO, and transmission electron microscopy (TEM) of (**c**) Gr (**d**) GO.

The scanning electron microscopy (SEM) and the electron transmission microscopy (TEM) were taken for both the Gr and the GO, which were found in Figure 8. Figure 8a,b showed the SEM for the Gr and GO samples. The images for the Gr structure (Figure 8a) indicated a layered morphology, an ordered sheetlike morphology. The SEM images of the GO (Figure 8b) showed slight transparency, indicating that the layers were smaller in number, but also it was seen rough and folded regions.

Images corresponding to transmission electron microscopy (TEM) spectra were also taken to analyze the material in detail on the surface of Gr and GO. It can be seen that monolayers have been easily isolated from the starting samples. In the images, it is shown how the structure of the monolayers was not completely flat, but rather that there were certain imperfections on the surface that caused irregularities in the plane (Figure 8c,d). These imperfections were closely related to the existence of sp^3^ hybridizing carbons that constituted hydroxyl or epoxy groups in the graphene oxide layers. In general, what the images indicated for Gr and GO was the presence of slightly wavy leaves, almost like “wrinkled leaves”, characteristics of Gr and GO, widely reported in the scientific literature [80,81,82,83,84,85,86]. This result verified what was found in analyses by other techniques so far discussed (XRD, Raman among others). When analyzing the GO TEM in detail, clearly folded sheets were observed spatially towards the edge. On the other hand, the TEM photomicrograph for GO revealed a clear image of the arrangement of the atom in a hexagonal shape, which confirmed the typical arrangement of the atom for graphene [84,85]. In summary, it has been demonstrated by various techniques that it was possible to synthesize graphene oxide from graphite. From this graphene oxide, its ability to adsorb Zn(II) and Pb(II) ions from aqueous solution will be investigated.

### 3.2. Analysis of N_2_ Adsorption Isotherms at 77 K

**Figure 9:**
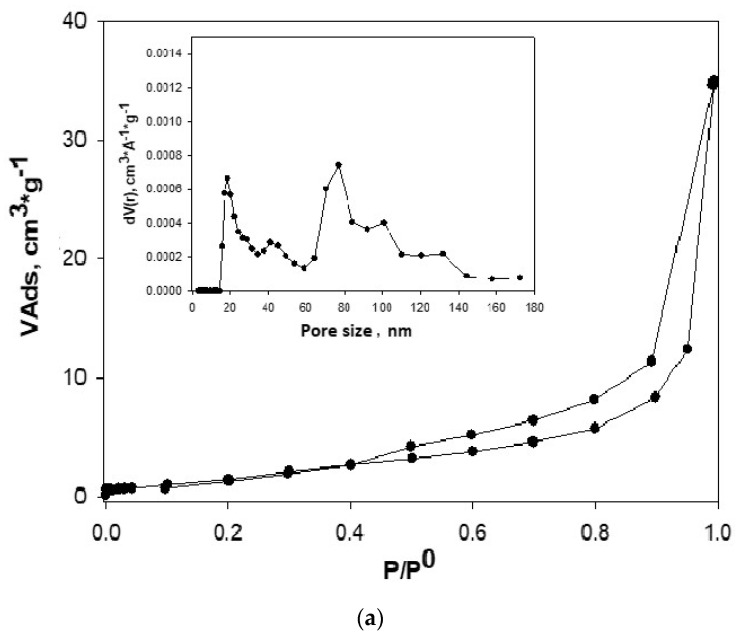

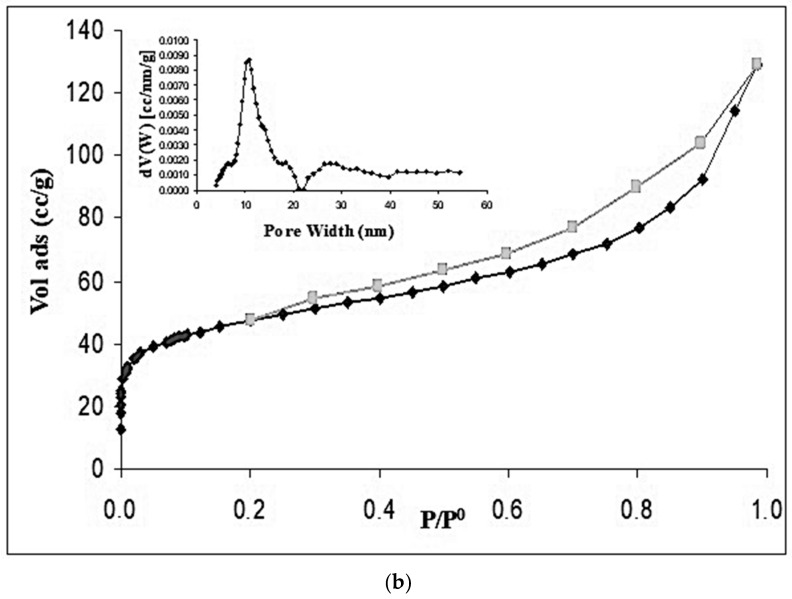
N_2_ gas adsorption-desorption isotherms of samples and pore size distribution (PSD) calculated with quenched solid density functional theory (QSDFT) kernel: (**a**) Gr (**b**) GO (**c**) simulation of Gr with nonlocal density functional theory (NLDFT) and QSDFT.

Figure 9 and Table 1 show the adsorption isotherms of N_2_ at 77 K and the textural parameters for both Gr and GO. These results allowed observing the changes introduced to Gr after being used as starting material for the synthesis of the GO. Figure 9 showed the N_2_ adsorption on the Figure 9a isotherm for graphite, which, according to the IUPAC (International Union of Pure and Applied Chemistry) in [86], is type IV. It had a hysteresis loop from P/P° at 0.40, which was type H4. For Figure 9b, the adsorption curve of the oxidized graphene mixture corresponded to type I and type IV isotherms, according to IUPAC classification, with a H3 hysteresis loop, which was presented from a P/P° of 0.5 [86,87]. The calculated PSD with the QSDFT (quenched solid density functional theory) kernel for the Gr and GO are in Figure 9c. Only the results of the distributions obtained with the QSDFT were shown because they better fitted the experimental data, as it can be seen in Table 2. The average pore width calculated by NLDFT and QSDFT kernels differ somewhat, although their magnitude was located within microporous materials, and specifically within the ultramicropores.

As can be seen, the average pore width calculated by the QSDFT (quenched solid density functional theory) kernel presented values of small pores, around 7.80 nm to 8.75 nm for Gr and GO respectively. This showed the change of Gr structure, compared to that of GO. This, together with the values obtained for the BET area as well as the micropore volumes (calculated by the DR method) and pore volumes calculated by using the DFT (density functional theory), showed structural and textural change.

The volumes found using the DR equation and the QSDFT and NLDFT models were consistent and very similar, which demonstrated the applicability of these models to both samples, Gr and GO. It was interesting to highlight this fact, since it was possible to use them in adsorption studies for interactions of these materials in the vapor phase and extend their applicability in the liquid phase. This type of correlation has not been reported in the literature until now, under the conditions of our research. In Table 2 we reported the results obtained by applying the NLDFT and QSDFT models assuming slit pores, cylinder and combined (cyl.-slit) for Gr and GO (to simplify Table 2, the data corresponding to pore volume were not published). The results of Table 2 presented data corresponding to % of error (E%) calculated from the mentioned kernels. Between the two kernels, QSDFT had the better fit because it had the lowest percentage error: 4.364% for Gr and 1.354% for GO, for slit pores—this despite being a model that fitted for the structure of both Gr and GO, if they are thought as laminar structures.

The modeling analysis indicated that the slit pore shape determined by the QSDFT kernel showed the best fit to the experimental data and this was in agreement with the results obtained experimentally with the isotherms of N_2_ at 77 K and that have been extensively analyzed before. This result indicated that the pores of these materials corresponded to ultramicroporous with rough and heterogeneous-energy surface. In Figure 9c, the adjustment of the experimental isotherms of Gr to NLDFT and QSDFT models was shown. It was clearly seen how the kernel of the QSDFT best fitted the experimental data. Something similar occurred for GO.

### 3.3. Analysis of the Adsorption Studies of Zn (II) and Pb (II) Ions on GO

#### 3.3.1. Effect of pH of Pb (II) and Zn (II) Ions on GO Surface

The pH variation in the study of the interaction in the adsorbate–adsorbent interface is important, not only because it allows establishing its influence on the charge and the state of the functional groups on the surface of the prepared GO. It also influences the state of the distribution curve of Pb (II) and Zn (II) ions when found in aqueous solution [63,64]. Determining the proper pH at which the maximum adsorption capacity is reached varies from one metal ion to another.

**Figure 10:**
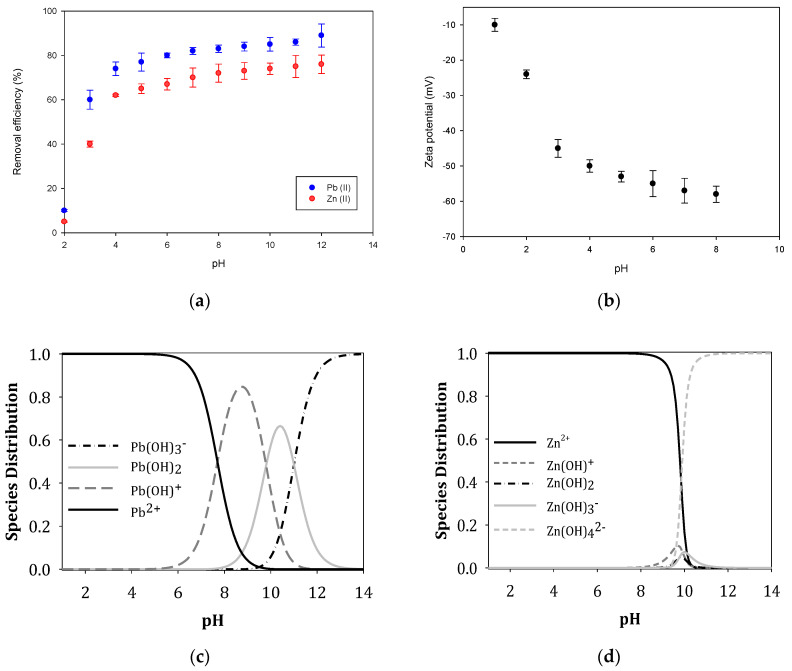
(**a**) Influence of pH on the adsorption of the metal ions Pb (II) and Zn (II) on GO (C_o_ = 5 mg L^−1^, C_GO_ = 0.5 mL^−1^, T = 25 C, t = 210 min). (**b**) Point zero charge. (**c**) Speciation graph of Pb. (**d**) Speciation graph of Zn.

In this study, a pH sweep was made from 2 to 10, to analyze in this pH range, the adsorption capacity of the solutions corresponding to the Pb(II) and Zn(II) ions on the GO, these results are shown in Figure 10. Previous experiments carried out to establish the equilibrium time; the results showed that after 210 min equilibrium was reached. When the metal ions are in aqueous solution they hydrate and form different complexes depending on the pH, which can be represented according to Equation (3) proposed by [86]:

(3) $$Me^{2+}↔Me{\left(OH\right)}^{+}↔Me{\left(OH\right)}_{2}↔Me{\left(OH\right)}_{3}^{-}↔\cdots$$

Figure 10a shows that at a pH between 2–3, the efficiency percentage in removing Pb (II) ions is low, which some authors [63,64,65,66,67,68,69,70] associate with the protonation of functional oxygen-containing groups in the GO surface. According to this argument, and considering that the GO surface in this pH range is positively charged, the electrostatic forces present prevented the Pb (II) ions from complexing, and the elimination percentage was low.

When the pH continued to increase until reaching a pH value of 5.5, the removal efficiency of the Pb(II) ions increased until reaching a value of 93%; this fact is associated with the oxygen-containing functional groups present weak interactions. At pH values above 6, a precipitation reaction was initiated, thus maintaining the removal efficiency at approximately 99%. In fact, the main states of lead in aqueous solution include Pb(II), Pb(OH)^+^, Pb(OH)_2_, Pb (OH)_3_^−^ and Pb(OH)^2−^_4_ [63,64]. When pH < 6, Pb(II) is the primary form. Therefore, a pH value of 5.5 was chosen for subsequent Pb(II) adsorption experiments.

When analyzing the effect of pH on the adsorption capacity for the Zn (II) ion from aqueous solution on the GO surface, the data reported in Figure 10 show that the adsorption of Zn (II) increases rapidly at pH values between 2.8–4.5 and thereafter remains constant around between pH 5.5–8.0. This means that in this pH range the species that is fundamentally present is like Zn (II) and has an adsorption capacity of >90% [86,87,88,89,90]. According to data reported in the scientific literature, the point zero charge for GO (pH_pzc_) is known to be between is 3.8–3.9 [63,64,65]. This means that, at pH >3.9, (pH > pH_pzc_), GO’s surface charge is fundamentally negative, and electrostatic interactions between metal ions and GO graphene sheets become stronger [86,87,88,89,90,91,92,93,94,95,96,97]. However, in this study, the point zero charge (pH_pzc_) was determined for GO. The results (see Figure 10b) showed that the GO surface was negatively charged at pH greater than 2. This explained (according to its speciation graph, see Figure 10c) why there was an increasing adsorption of Zn ions on GO surface at pH between 2.0–7.0, which could be attributed to the electrostatic attraction between the Zn(II) ions and the negative charge of GO. The significant percentage of Zn(II) elimination at pH 7.0–9.0, could be attributed to the simultaneous precipitation of Zn(OH)_2_, which was in agreement with the previous study. At a higher pH, 9.0, the electrostatic repulsion between the GO and Zn (OH)^3−^ and negatively charged Zn (OH)_4_^2−^ species resulted in a slight reduction in the adsorption of Zn(II) adsorption on GO.

In the case of lead, its speciation graph (see Figure 10d) was remarkably different compared to the one of zinc, this was why its adsorption capacity changed. In the first part of the pH range, lead adsorption increased considerably due to the attraction of the negative charge on the GO surface with the positive charge of the Pb(II) ion. Subsequently, after a pH of approximately 8.5, the amount of adsorption changed because the predominant species was Pb(OH)_2_. In summary, when pH < pH_pzc_, the surface charge of the adsorbent was positive due to the protonation reaction (SOH^+^ H^+^ → SOH_2_^+^). On the another hand, if pH > pH_pzc_, the surface charge of the adsorbent was negative due to the deprotonation reaction (SOH→SO^−^ + H^+^), where S represented the surface of the adsorbent, and –OH represented the oxygen functional groups [98,99].

#### 3.3.2. Analysis of Results of the Adsorption Isotherms for Pb(II) and Zn(II) Ions on GO

The analysis of the results of the adsorption isotherm of the ions on the GO from aqueous solution, allows us to know the relationship between the concentration of the adsorbate and the degree of adsorption on the surface of the adsorbent at a constant temperature. To establish the adsorption capacity of graphene oxide in removing Pb(II) and Zn(II) ions from aqueous solution, (widely used) models were applied to the experimental data of the isotherm to know the best fit to one of these and calculate the adsorption parameters of each model. The applied models are: Langmuir, Freundlich, Dubinin–Radushkevich (DR) and Temkim. The models were applied to simulate and understand the mechanism of adsorption of metal ions Pb(II) and Zn(II) by adsorbing on the graphene sheets that make up the structure of graphene oxide (GO). The equations that represent these models and the meaning of each of their parameters are shown below.

##### Langmuir Model

It is based on the assumption that the adsorbent has a uniform adsorption energy at a constant temperature during the formation of a monolayer by the adsorbed solute. The linear form is expressed in Equation (4) [100,101].

(4) Ceqe=Ceqm+1(KLqm)

where q_e_ and ce are the equilibrium concentrations of Pb (II) and Zn (II) ions in the adsorbed (mg·g^−1^) and liquid (mg·mL^−1^) phases, respectively. q_m_ and K_L_ are the Langmuir constants, which can be calculated from the graph, C_e_/q_e_ versus Ce. The Langmuir isotherm separating factor is expressed in terms of the K_L_ parameter. It is a dimensionless constant. The K_L_ value shows the behavior of the process. If K_L_ > 1, it is unfavorable, if K_L_ = 1, it is linear, if 0 < K_L_ <1 is favorable, and it is irreversible if K_L_ = 0.

##### Freundlich Model

In this model, it assumes that adsorption occurs on a heterogeneous surface with uniform energy and in multilayer, and can be represented in its linear form by Equation (5) [102]:

Q_e_ = K_F_ × C_e_^1/n^ (5)

q_e_ is the amount adsorbed per unit mass of adsorbent (mg/g), C_e_ is the concentration of adsorbate in equilibrium in solution, K_F_ (mg^1−n^ L^n^ g ^−1^) and n are the Freundlich constants related to the adsorption capacity and the adsorption intensity, respectively.

##### Dubinin–Radushkevich (DR) Isotherm Model

Applying this empirical model (DR) allows the establishing of whether the adsorption is of a physical or chemical nature [103,104]. The DR model is presented in Equation (6), where K_E_ is a constant related to the adsorption energy (mol^2^·KJ^−2^) and Q_m_ is the theoretical constant DR (mg·g^−1^). ε is the Polanyi potential and is calculated from the Equation (7), where R is the molar gas constant (J·mol^−1^·K^−1^) and T is the absolute temperature (K). The Q_m_ and K_E_ values are obtained from the linear graph of ln q_e_ vs. ε^2^ (Table 1). The mean adsorption energy, E (kJ·mol^−1^), can be obtained by applying the K_E_ value of the isotherm equation DR in the Equation (8). E, provides useful information on the type and/or mechanism of the adsorption process. If E is less than 8 kJ/mol, the adsorption is physical in nature; if it is between 8 and 16 kJ/mol, the adsorption process is carried out by means of ion exchange. If E is greater than 16 kJ/mol, the mechanism of particle diffusion and chemical reaction will dominate the process [59,60,61]. Therefore, from this result, it is concluded according to the results reported in Table 1, that the adsorption process is chemisorption.

(6) $$\ln q_{e}=\ln Q_{m}-K_{E}\varepsilon^{2}$$

(7) $$\varepsilon =\mathrm{RT}\;\ln\left[1+\frac{1}{C_{e}}\right]$$

(8) $$E={\left(-2K_{E}\right)}^{-\left(1/2\right)}$$

##### Temkin Model

Temkin’s model allows us to analyze the interactions between ions and GO, and the energy of adsorption. In Temkin’s model, adsorption is characterized by a uniform distribution of the binding energy, up to a maximum binding energy [105], which supposes that the decrease in the heat of absorption is linear instead of logarithmic, as occurs in the equation Freundlich [75]. The Temkin isotherm can be applied as given in Equation (9), where K_T_ is the equilibrium bonding constant (L·mol^−1^) corresponding to the maximum bonding energy, b is related to the heat of adsorption, R is the universal gas constant (8314 JK^−1^·mol^−1^) and T is the temperature (K):

(9) $$q_{e}=\frac{\mathrm{RT}}{b}\ln K_{T}+\frac{\mathrm{RT}}{b}\ln C_{e}$$

The parameters for each of the isotherm models used are presented in Table 1. From the results, it is observed that the experimental adsorption data fit the Langmuir model better, bearing in mind that it has the highest R^2^ (0.996 and 0.964 for Pb(II) and Zn(II) respectively), which shows that the adsorption of the metal ions Pb(II) and Zn(II) on the GO graphene sheets occurs through the formation of a monolayer. This is possible, if the structure of the GO are layers where when the ions enter between them they only reach to form a single layer.

The Q_m_ values for adsorption of Pb(II) and Zn(II) are 987.33 and 313.43 mg·g ^−1^, respectively. Furthermore, the K_L_ values are in the range 0 < K_L_ < 1, which describes adsorption as a favorable process. When comparing the difference in affinity of GO for the two study ions in this study and their electronegativity variable, it is observed that the adsorption is Pb (II) > Zn (II). According to what was reported by other authors [83,84] this order agrees very well with the electronegativity of the metal and the first constant of stability of the associated metal hydroxide [83]. The adsorption of metal ions can be interpreted like complexation with functional surface groups (e.g., -OH and -COOH). For this reason, it has been published that the stability constant of metal ions may be a relevant correlation parameter [63]. The order of affinity agrees well with the first stability constant of the associated metal hydroxide (Me^2+^ + OH− ↔ Me (OH)^+^; log K_1_ = 7.82 and 4.40 for Pb (OH)^+^ and Zn (OH)^+^, respectively) [83,106]. This is additionally associated with the extremely hydrophilic capacity and the presence of functional groups containing oxygen atoms in the GO, which explains the high adsorption capacity of the ions.

The Dubinin–Radushkevich model [97] can explain the nature of the adsorption process, i.e., whether it proceeds via chemisorption or physisorption. According to this model, when the value of E was higher than 8 kJ·mol^−1^, the adsorption process could be considered a chemisorption. The values of E, which are shown in Table 3, were lower than 8 kJ·mol^−1^, leading to the conclusion that the investigated adsorption was physical in nature, for the case of the adsorption of Pb(II) and Zn(II) ions on GO. The correlation coefficients (R^2^) were 0.9462 and 0.9571 for Pb(II) and Zn(II) ions, respectively, for the Dubinin–Radushkevich model, because of this, the application of this model in this research was limited.

**Figure 11:**
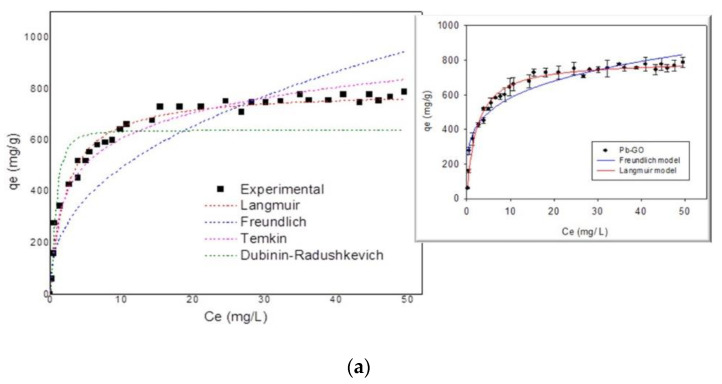

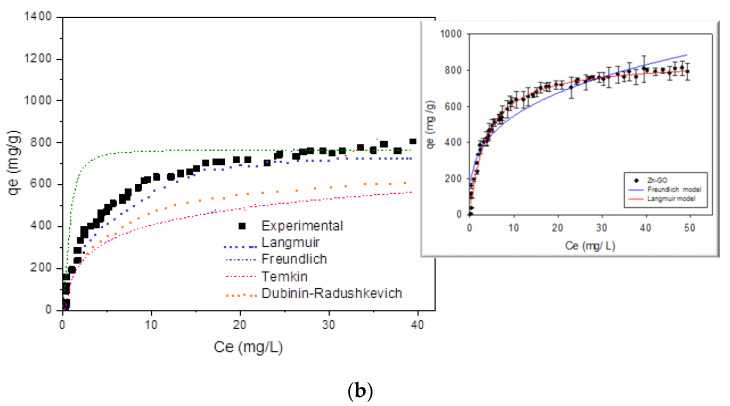
Adsorption isotherms of Pb(II) and Zn(II) ions on GO: (**a**) Pb on GO (**b**) Zn on GO.

Figure 11 shows the results of the isotherms studied and their trend lines, which confirm that the Langmuir model is the one that best fits the experimental data.

The adsorption capacities obtained for lead and zinc were compared against carbon-based adsorbents [18,60,61,62,63,64,65,66] and other porous solids. According to results, the graphene oxide synthesized in this research showed excellent adsorption capacity, as is evident from the data presented in Table 4; it even surpasses some adsorbents consulted. So, it is evident that graphene oxide is an effective adsorbent for eliminating Pb(II) and Zn(II) from effluents. These adsorption capacities are additionally associated with heterogeneity of energetic type developed on the surface of GO during synthesis, as it was demonstrated in the QSDFT analysis.

#### 3.3.3. Studies of the Adsorption Kinetics of Pb (II) and Zn (II) Ions on GO

**Figure 12:**
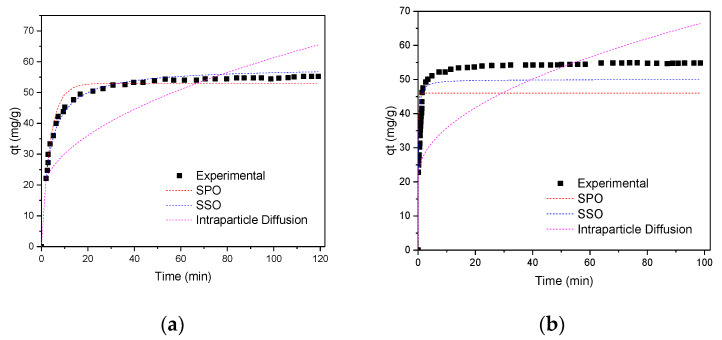
Adsorption kinetics on GO. Experimental conditions: Co = 25 mg L^−1^, pH = 5.5, T = 298 K, CGO = 1.0 mg mL^−1^: (**a**) Pb (II) on GO (**b**) Zn (II) on GO. SPO: pseudo-primer order; SSO: pseudo-second Order.

The results obtained from the behavior of the elimination capacity of the Pb (II) and Zn (II) ions, as a function of the time variable, are presented in Figure 12 (the adjustments are presented for the pseudo-first order, pseudo-second order, intraparticle diffusion (Weber–Morris) and Elovich equation—this is not shown, because the experimental results did not fit well. Carrying out kinetic studies of adsorbate–adsorbent interactions is a very important aspect when investigating the adsorption process, because according to the results obtained, the applicability of the adsorbent can be greatly restricted or benefited. If we have a process where the adsorption kinetics are slow, this causes the ion adsorption time not to be adequate [122,123]. Additionally, it is known that the adsorption mechanism depends on the physical and chemical characteristics of the adsorbent [83]. Therefore, in this investigation, several models were applied to establish the kinetic order of the adsorption of Pb(II) and Zn(II) ions on the GO surface.

The results obtained from the experimental data for kinetics were adjusted to the following models: pseudo-first order, pseudo-second order, intraparticle diffusion (Weber–Morris) and Elovich equation. The linearized equation for the pseudo-first-order expression (Lagergren) is given by the following expression represented by Equation (10) [124,125,126,127]:

(10) $$\log\left(q_{e}-q_{t}\right)=\log q_{e}-\frac{k_{1}}{2.303}t$$

where q_e_ and q_t_ correspond to the quantity of the ions Pb (II) and Zn (II) adsorbed at equilibrium and at time t (mg/g), respectively. The adsorption rate constant k_1_ (min^−1^) can be calculated from the linear representation of log (q_e_ − q_t_) as a function of t.

The results were also modeled according to the pseudo-second-order kinetics expressed as according to Equation (11) [126]:

(11) $$\frac{t}{q_{t}}=\frac{1}{k_{2}q_{e}^{2}}+\frac{t}{q_{e}}$$

where k_2_ (g/mg.min) corresponds to the equilibrium speed constant of the pseudo-second-order kinetic model. The values of k_2_ can be determined from the graph between t/q_t_ as a function of t.

On the other hand, it is also possible to study the adsorption kinetics from a mechanistic point of view. An adsorption process according to the system under study, can be controlled by one or more steps, e.g., by a film or external diffusion, pore diffusion, surface diffusion and pore surface adsorption, or a combination of more than one step [88]. In general, in a porous system, a process can be controlled by diffusion of the adsorbate towards the adsorbent if its speed depends on the speed at which the components diffuse from each other. The possibility of intraparticle diffusion was explored using the Weber–Morris model [127,128,129,130,131]. The Weber–Morris model is expressed by Equation (12) [73,128,129]:

(12) $$q_{t}=K_{I}t^{\frac{1}{2}}+C$$

in this expression K_I_ is the intraparticle diffusion rate constant (mg g min^1/2^) and is obtained from the slope of the corresponding graph. To prove the existence of intraparticle diffusion in the adsorption process, the amount of Pb(II) and Zn(II) adsorbed, q_t_, at any time t (mg/g), was represented as a function of the square root of time (t^1/2^).

The adsorption data was also analyzed using the Elovich equation, which has the following equation linearly (13) [73,130]:

(13) $$q_{t}=\left(\frac{1}{\beta }\right)\ln\left(\alpha \beta \right)+\left(\frac{1}{\beta }\right)\ln\left(t\right)$$

where α is the constant of the initial adsorption rate (mmol/gmin), and the parameter β is related to the scope of the surface coverage and the activation energy for the chemisorption (g/mmol) [73].

As can be seen in Figure 12a,b, the adsorption for both Pb(II) and Zn(II) on GO, increased rapidly at the beginning of each experiment, and then reached a plate, which is characteristic when the system reaches the equilibrium. However, the times are different for each ion. According to these results, it is inferred that the balance depends on the nature of the ions: the percentage of adsorption for the Pb (II) ion was reached after 20 min, achieving the GO removal of 90%, while for the Zn(II) ion, this same removal percentage is reached in just 3 min. The 98% adsorption was achieved after 80 min for Pb (II), and after 30 min for Zn (II) [87,88].

From the graphs of the kinetic studies represented in Figure 10, these were linearized. From these representations (not included in this work), in addition to performing a residual analysis (not presented here either) of each of the graphs and establishing that none presented any trend, it was established that the correlation coefficients were the appropriate parameters to evaluate which was the best kinetic model that described the adsorption of Pb(II) and Zn(II) ions on GO. When analyzing the results of Table 5, it can be seen that the best R^2^ was presented, for the two ions, by the pseudo-second order kinetic model with a value of 0.999. This means that the process of adsorption of ions on the GO surface involves a chemisorption mechanism, with a complexing reaction of the adsorbates on the surface of the adsorbent (GO). This result allows us to infer that the adsorption capacity is proportional to the number of active sites occupied on the GO surface [63,83,84,85,86,87,88,89,90,91,92,93,94,95]. When comparing the four graphs that represent the linear models, it is observed that they all increase as a function of time, except for the pseudo-first-order model, which decreased.

When the results were adjusted to the kinetic model of intraparticle diffusion, these had a good fit since the R^2^ values were of 0.937 and 0.943 for Pb(II) and Zn(II) respectively. The values of C (which represented the effect of the boundary layer in this model) presented small values: 28.8 (Pb (II)) and 23.8 (Zn (II)); finally, values were obtained for K_I_ (the rate constant) of 0.453 and 0.415 (Pb(II) and Zn(II), respectively). These values of the rate constants were less than 1, which showed that the velocity of the ions to the internal structure of graphene oxide was not favored. [86,125,126,127,128,129,130,131]. These results allowed us to conclude that the intraparticle diffusion kinetic model was not the determining step of the velocity for the ion adsorption processes having a C intercept that was > 0 <1. With the graph t^1/2^ vs. q_t_, the plot presented two different parts, indicating the different stages in adsorption (not shown here). The first part represented the external mass transfer. The second one was the gradual adsorption stage where intraparticle diffusion was rate-limiting. As the lines did not pass through the origin, this indicated that the intraparticle diffusion was involved in the adsorption process but it was not the only rate-controlling step. The values of C were helpful to determine the boundary thickness: a larger C value corresponded to a greater boundary layer diffusion effect. Therefore, the kinetics were affected by some other adsorption processes in the system as shown by the value of β obtained in the Elovich model, that according to the correlation coefficient values, it could be concluded that the Elovich model described chemically the adsorption of Pb(II) and Zn(II) onto the GO adsorbent [125,126,127,128,129,130,131].

#### 3.3.4. Analysis of Thermodynamic Parameters in the Adsorbate–Adsorbent System

Studying thermodynamics in an adsorption process provides information about the nature of the process; thus, the change in Gibbs free energy (∆G^o^), entropy (∆S^o^) and standard enthalpy (∆H^o^), were determined from the following Equation (14): [61,62,63,64,65,66,67,68,69,70,71]:

(14) $$\Delta G^{o}=-\mathrm{RT}\ln K_{o}=\Delta H^{o}-T\Delta S^{o}$$

where R is the universal gas constant, and T is the absolute temperature in K. K_o_ is the thermodynamic equilibrium constant in mg/L, which can be calculated by q_e_/C_e_. From Equation (13), the standard enthalpy change, ΔH^o^, and the standard entropy change, ΔS^o^, can be obtained from the graph of lnK_o_ as a function of 1/T using Equation (15):

(15) $$\ln K_{o}=\frac{\Delta S^{o}}{R}-\frac{\Delta H^{o}}{\mathrm{RT}}$$

These thermodynamic variables establish the spontaneity of a given process at a certain temperature. When the adsorbate and adsorbent come into contact, an equilibrium is reached after a certain time, which can be described by adsorption isotherms. Table 6 shows the results obtained for each of the thermodynamic variables evaluated in this investigation with the results obtained.

According to the results shown in Table 6, the negative Gibbs free energy values (ΔG^o^) suggest that the adsorption of Pb(II) and Zn(II) ions on the GO adsorbent is thermodynamically spontaneous and feasible. Furthermore, the high negative ΔG^o^ values of the ion–GO system (see Table 3) as the temperature increases confirm that the adsorption of these metal ions is favorable at higher temperatures and that it can be caused by electrostatic repulsions between the adsorbate and the surface of the adsorbent and to the mobility of the metal ions towards the active sites of the adsorbent (GO). As discussed by some authors in the scientific literature [90,91,92,93,94,95,96,97,98], the nature of adsorption can be classified as processes of chemisorption and fisisorption when the ΔG^o^ values are in the ranges −80 to −400 kJ mol^−1^ and 0–20 kJ mol^−1^, respectively. According to the results obtained, the adsorption of metal ions on graphene oxide is a physical adsorption process. The positive values obtained for ΔH^o^ for the ion-GO system indicate that the nature of the studied process is endothermic. To explain this behavior (GO) they are favorable at high temperatures because all metal ions are dehydrated and their solubility in the aqueous solution increases with increasing temperature [128,129,130,131]. Furthermore, the positive ΔS^o^ values reflect the affinity of the GO adsorbent towards metal ions in aqueous solutions and an increase in the disorder at the solid–solution interface during the adsorption process [63,90,91,92,93,94,95,96,97,98].

## 4. Conclusions

In this research, it was possible to synthesize graphene oxide (GO) from graphite oxidation (Gr). Different techniques were used to demonstrate the obtaining of GO. The high adsorption capacity of metal ions on GO was found to be due to their hydrophilic properties and the presence of functional groups containing oxygen atoms. These groups can efficiently bond metal ions to form a metal complex because they share an electron pair. The synthesized GO has maximum adsorption capacities towards Pb(II) and Zn(II), of 987.33 and 313.43 mg g^−1^ respectively. The adsorption of Pb (II) and Zn(II) shows that GO’s affinity for these metal ions follow the order of Pb (II) > Zn (II). The adsorption isotherms conform to the Langmuir model, which means that the adsorption of metal ions in the GO graphene sheets occurs with a monolayer coating. The kinetic study indicates that the adsorption of metal ions on GO is controlled by chemical adsorption (chemisorption), which involves the superficial complexation of metal ions with oxygen-containing groups on the GO surface, since it was fitted to the pseudo-second-order kinetic model. Finally, the thermodynamic analysis shows that the Gibbs free energy (ΔG^o^) suggest that the adsorption of Pb(II) and Zn(II) ions on the GO adsorbent is thermodynamically spontaneous and feasible. The pores of samples were modeled from the N_2_ adsorption isotherms data at 77 K, using NLDFT kernels. The surface of Gr and GO corresponded to a slit-type pore according to QSDFT model.

## Figures and Tables

**Table 1:** Dubinin–Radushkevich (DR) and functional theory of density (DFT) parameters obtained from the adsorption-desorption isotherms of N_2_ at 77.4 K.

Samples	S_BET_ [m^2^·g^−1^]	DR (P/P° < 0.1)				DFT (P/P° 10^−7^ − 1)	
		V_mic_ [cm^3^·g^−1^]	E_o_ [Kj·mol^−1^]	n	Pore Radius [Å]	V_P_ [cm^3^·g^−1^]	Half Pore Width [nm]
Graphite (Gr)	5.2	0.010	7.250	3.4	7.4	0.04	7.80
Oxide Graphene (GO)	47.5	0.154	18.60	5.4	9.3	0.18	8.75

**Table 2:** Fitting error for different surface textures (NLDFT vs. QSDFT) in slit and slit/cylindrical pores.

	NLDFT			QSDFT		
Sample	Fitting Error (Slit Pore) [%]	Fitting Error (Cyl. Pore) [%]	Fitting Error (Cyl.-Slit) [%]	Fitting Error (Slit Pore) [%]	Fitting Error (Cyl. Pore) [%]	Fitting Error (Cyl.-Silt) [%]
Graphite (Gr)	5.296	6.819	6.819	4.364	7.510	7.910
Graphene Oxide (GO)	2.345	3.576	4.765	1.354	2.546	3.087

**Table 3:** Equilibrium adsorption isotherms parameters for Pb (II) and Zn (II) on GO.

Adsorption Parameters	Metals Ions	
	Pb (II)	Zn (II)
Langmuir model		
Q_m_ (mg·g^−1^)	987.3	313.4
K_L_ (L·mg^−1^)	0.1237	0.1035
R^2^	0.9968	0.9989
Freundlich model		
K_F_ (mg·g^−1^) (L·mg^−1^)^1/n^	290.5	96.56
n	4.1672	
R^2^	0.9647	
Dubinin–Radushkevich model		
Q_m_ (g·g^−1^)	123.4	95.32
E (kJ·mol^−1^)	6.5301	4.3021
R^2^	0.9461	0.9570
Temkin model		
K_T_ (L·mol^−1^)	0.9651	0.3722
b (J·g·mol^−2^)	645.3	321.3
R^2^	0.8310	0.7622

**Table 4:** Comparison of adsorption capacities with other adsorbents involving graphene oxide and other related materials.

Adsorbent	Pb(II) mg·g^−1^	Zn(II) mg·g^−1^	Ref.
Aloji clay	39. 30		[107]
Pillared clays	222.22		[108]
Activated carbon prepared from palm oil mill effluent New	69.44	59.88	[109]
carbon-doped ferric zinc oxide	150.0		[110]
Aspergillus flavus biomass		27.855	[111]
Purolite C100-MH resin	64.10		[112]
Graphite doped chitosan composite	6.711		[113]
Magnetic graphite	38.5		[114]
Carbon nanotubes		32.68	[115]
Graphene Oxide		246.0	[116]
Nanoporous carbon		130.76	[117]
Graphene oxide	250.0		[118]
Few-layered graphene oxide	842.0		[119]
Graphene oxide		246.0	[120]
Graphene oxide		345.0	[121]
Graphene oxide	987.33	313.43	Present study

**Table 5:** Determined kinetic parameters for the adsorption of Pb(II) and Zn(II) on GO, Conditions: pH = 5.5; T = 298 K; into an aqueous solution of initial concentration 25 mg L^−1^.

Model	Parameters	Adsorbate	
		Pb (II)	Zn (II)
Pseudo-first order	q_e_ (mg/g)	196.7	169.6
	K_1_ × 10^4^ (1/min)	4.5406	2.3402
	R^2^	0.9560	0.9341
Pseudo-second order	q_e_ (mg/g)	220.5	199.6
	K_2_ (g/mg min)	0.2450	0.1351
	R^2^	0.9998	0.9998
Elovich	α (mmol·g^−1^·min^−1^)	189.3	152.1
	β × 104 (mmol·g^−1^)	4.0232	2.1005
	R^2^	0.9878	0.9768
Intraparticle diffusion	C (mg/g)	28.84	23.82
	K_I_ (mg g min^1/2^)	0.4532	0.4151
	R^2^	0.9375	0.9436

**Table 6:** Thermodynamics parameters for Pb (II) and Zn (I) ions adsorption on GO adsorbent.

Metal Ions	Temperature (K)	(ΔG^o^) (kJ mol^−1^)	(ΔS^o^) (JK^−1^ mol^−1^)	(ΔH^o^) (kJ mol^−1^)	R^2^
Pb (II)	298	−6787	45.32	37.54	0.995
	308	−7165			
	318	−7784			
Zn (II)	298	−5497	32.48	23.12	0.997
	308	−5871			
	318	−6187			
